# Combined metabolic activators improve cognitive functions in Alzheimer’s disease patients: a randomised, double-blinded, placebo-controlled phase-II trial

**DOI:** 10.1186/s40035-023-00336-2

**Published:** 2023-01-26

**Authors:** Burak Yulug, Ozlem Altay, Xiangyu Li, Lutfu Hanoglu, Seyda Cankaya, Simon Lam, Halil Aziz Velioglu, Hong Yang, Ebru Coskun, Ezgi Idil, Rahim Nogaylar, Ahmet Ozsimsek, Cemil Bayram, Ismail Bolat, Sena Oner, Ozlem Ozdemir Tozlu, Mehmet Enes Arslan, Ahmet Hacimuftuoglu, Serkan Yildirim, Muhammad Arif, Saeed Shoaie, Cheng Zhang, Jens Nielsen, Hasan Turkez, Jan Borén, Mathias Uhlén, Adil Mardinoglu

**Affiliations:** 1 Department of Neurology and Neuroscience, Faculty of Medicine, Alanya Alaaddin Keykubat University, Antalya, Turkey; 2 Science for Life Laboratory, KTH - Royal Institute of Technology, Stockholm, Sweden; 3 Department of Neurology, Faculty of Medicine, Istanbul Medipol University, Istanbul, Turkey; 4 Centre for Host-Microbiome Interaction’s, Faculty of Dentistry, Oral and Craniofacial Sciences, King’s College London, London, UK; 5 Department of Women’s and Children’s Health, Karolinska Institute, Stockholm, Sweden; 6 Functional Imaging and Cognitive-Affective Neuroscience Lab, Istanbul Medipol University, Istanbul, Turkey; 7 Department of Medical Pharmacology, Faculty of Medicine, Atatürk University, Erzurum, Turkey; 8 Department of Pathology, Veterinary Faculty, Ataturk University, Erzurum, Turkey; 9 Department of Molecular Biology and Genetics, Faculty of Science, Erzurum Technical University, Erzurum, Turkey; 10 School of Pharmaceutical Sciences, Zhengzhou University, Zhengzhou, People’s Republic of China; 11 Department of Biology and Biological Engineering, Chalmers University of Technology, Gothenburg, Sweden; 12 Department of Medical Biology, Faculty of Medicine, Atatürk University, Erzurum, Turkey; 13 Department of Molecular and Clinical Medicine, University of Gothenburg and Sahlgrenska University Hospital, Gothenburg, Sweden

**Keywords:** Alzheimer’s disease, Combined metabolic activators, Multi-omics, Systems biology, Systems medicine

## Abstract

**Background:**

Alzheimer’s disease (AD) is associated with metabolic abnormalities linked to critical elements of neurodegeneration. We recently administered combined metabolic activators (CMA) to the AD rat model and observed that CMA improves the AD-associated histological parameters in the animals. CMA promotes mitochondrial fatty acid uptake from the cytosol, facilitates fatty acid oxidation in the mitochondria, and alleviates oxidative stress.

**Methods:**

Here, we designed a randomised, double-blinded, placebo-controlled phase-II clinical trial and studied the effect of CMA administration on the global metabolism of AD patients. One-dose CMA included 12.35 g *L*-serine (61.75%), 1 g nicotinamide riboside (5%), 2.55 g N-acetyl-*L*-cysteine (12.75%), and 3.73 g *L*-carnitine tartrate (18.65%). AD patients received one dose of CMA or placebo daily during the first 28 days and twice daily between day 28 and day 84. The primary endpoint was the difference in the cognitive function and daily living activity scores between the placebo and the treatment arms. The secondary aim of this study was to evaluate the safety and tolerability of CMA. A comprehensive plasma metabolome and proteome analysis was also performed to evaluate the efficacy of the CMA in AD patients.

**Results:**

We showed a significant decrease of AD Assessment Scale-cognitive subscale (ADAS-Cog) score on day 84 vs day 0 (*P* = 0.00001, 29% improvement) in the CMA group. Moreover, there was a significant decline (*P* = 0.0073) in ADAS-Cog scores (improvement of cognitive functions) in the CMA compared to the placebo group in patients with higher ADAS-Cog scores. Improved cognitive functions in AD patients were supported by the relevant alterations in the hippocampal volumes and cortical thickness based on imaging analysis. Moreover, the plasma levels of proteins and metabolites associated with NAD + and glutathione metabolism were significantly improved after CMA treatment.

**Conclusion:**

Our results indicate that treatment of AD patients with CMA can lead to enhanced cognitive functions and improved clinical parameters associated with phenomics, metabolomics, proteomics and imaging analysis.

*Trial registration* ClinicalTrials.gov NCT04044131 Registered 17 July 2019, https://clinicaltrials.gov/ct2/show/NCT04044131

**Supplementary Information:**

The online version contains supplementary material available at 10.1186/s40035-023-00336-2.

## Background

Alzheimer’s disease (AD) is characterised by progressive synaptic and axonal dysfunction, neuronal loss and cognitive decline [1]. There is growing evidence that AD is closely associated with metabolic abnormalities and oxidative stress linked to critical elements of neurodegeneration, such as mitochondrial dysfunction and bioenergetic impairment [2, 3]. Indeed, increasing data indicate that systemic metabolic disorders, such as insulin resistance, are strongly associated with bioenergetic failure of nerve cells [4, 5]. This can manifest as cognitive impairment and brain-specific neuropathology, and share common pathogenic mechanisms with AD, such as impaired glucose metabolism, oxidative stress, insulin resistance, and amyloidogenesis [4, 6, 7]. Recent evidence suggests that patients with type 2 diabetes mellitus are at an increased risk of developing AD [6].

Although AD is defined by accumulation of abnormal amyloid and tau proteins [8], the mechanistic assumption of linear causality between the amyloid cascade and cognitive dysfunction in AD is still lacking, since amyloid-lowering approaches have failed to provide cognitive benefits in human clinical trials [9]. A growing body of evidence suggests that impaired brain energy metabolism and mitochondrial dysfunction in AD may contribute to cognitive decline. At the same time, different drugs for metabolic diseases are prescribed to improve metabolic status, slow cognitive decline or prevent dementia progression [10]. Positron emission tomography studies have revealed baseline cerebral glucose metabolism abnormalities before the onset of cognitive symptoms in AD patients [11]. In addition, recent preclinical data indicated that ageing and AD are associated with the reorganisation of brain energy metabolism and mitochondrial dysfunction, including an overall increase in lactate secretion and downregulation of bioenergetic enzymes [12, 13].

Several studies have suggested that impaired brain energy metabolism and oxidative stress are associated with mitochondrial degeneration and abnormal protein accumulation during the progress of AD [14–18]. In this context, emerging evidence suggests that the autophagy/lysosome pathways play a critical role in removing damaged mitochondria (mitophagy), and dysfunction of autophagy results in the accumulation of dysfunctional mitochondria in neurons [19]. This opens up a new window of beneficial effects of interventions that maintain mitochondrial health and/or stimulate mitophagy in the neurodegenerative process in AD [20]. In line with this, the beneficial effects of nicotinamide on mitochondrial integrity, autophagy and bioenergetics-related signaling in brain cells are associated with reduced accumulation of abnormal Aβ and tau in the hippocampus, and lead to the improved cognitive performance in transgenic mice [21, 22]. Also, recent human studies suggested that the impaired hippocampal mitophagy in AD patients responds well to mitophagy enhancement strategies and such treatment finally improves AD-related tau pathologies in human neuronal cells and memory deficits in transgenic models [23].

Combining multiple compounds to both reduce oxidative injury and improve bioenergetics, in other words, to target multiple pathways simultaneously, has been proposed as a therapeutic strategy associated more likely with successful translational outcomes [24]. Previous research identified limited serine availability, reduced de novo glutathione (GSH) synthesis, and altered NAD+ metabolism in a transgenic mouse model of AD based on multi-omics profiling [25]. While NAD+ is reduced in the AD animal models, NAD+ augmentation mitigates Aβ, tau, and metabolic pathologies in laboratory models of AD [23, 26]. These findings have been confirmed by human metabolomic data showing significantly altered cerebrospinal fluid levels of acylcarnitine in patients with AD, which are correlated with the decline of cognitive function and structural abnormalities of the brain [27, 28].

In addition to the above-mentioned metabolic underpinnings of AD, several neuroimaging studies have revealed alterations of critical cognitive regions, including hippocampus, cortex, and inferior parietal, middle frontal and occipital regions [29]. For instance, Nagata et al. showed that the vulnerability of the hippocampus plays a potential role in memory and executive dysfunction in AD patients [30, 31]. Similarly, several neuroimaging studies showed that cortical thickness plays a critical role in AD pathophysiology [32, 33]. Despite these promising studies, no research has evaluated the common pathophysiological mechanisms shared by systemic metabolic alterations and specific brain areas involved in cognitive deterioration in AD patients.

Based on the integrative network analysis of multi-omics data of non-alcoholic fatty liver disease, we have developed the combined metabolic activators (CMA) consisting of *L*-serine, *N*-acetyl cysteine (NAC), nicotinamide riboside (NR), and *L*-carnitine tartrate (LCAT, the salt form of *L*-carnitine) and showed that administration of CMA activates mitochondria, and improves inflammatory markers in animals and humans [34–38]. We have found that the CMA administration promotes mitochondrial fatty acid uptake from the cytosol, facilitates fatty acid oxidation in the mitochondria, and alleviates oxidative stress [39]. Global metabolomic and proteomic profiling revealed that CMA administration effectively increases fatty acid oxidation and de novo GSH synthesis [34]. Moreover, plasma levels of metabolites associated with antioxidant metabolism and inflammation are improved in COVID-19 patients treated with CMA compared to the placebo [38]. Recently, we tested the individual metabolic activators and CMA in the streptozotocin-induced AD-like rats and showed that CMA administration significantly improved behavioural scores in parallel with neurohistological outcomes in this model [40].

Based on these studies, we hypothesized that CMA administration may be a promising treatment for improving the metabolic parameters and brain functions in AD patients. Here, we designed a randomised, double-blinded, placebo-controlled human phase 2 clinical study to investigate the effect of CMA administration on the global metabolism of AD patients through comprehensive phenomics, metabolomics, proteomics and imaging analyses.

## Materials and methods

### Clinical trial design and oversight

Patients for this randomised, parallel-group, two-arm, double-blinded, placebo-controlled, phase 2 study were recruited at the Faculty of Medicine, Alanya Alaaddin Keykubat University, Antalya, Turkey and Faculty of Medicine, Istanbul Medipol University, Istanbul, Turkey. Written informed consent was obtained from all participants before initiating any trial-related procedures. An independent external data-monitoring committee oversaw the safety of the participants and the risk–benefit analysis. The trial was conducted following Good Clinical Practice guidelines and the principles of the Declaration of Helsinki. This study was approved by the ethics committee of Istanbul Medipol University, Istanbul, Turkey (Date:22.01.2020, Decision No: 7), and registered at https://clinicaltrials.gov/ with Clinical Trial ID: NCT04044131.

### Eligibility criteria of clinical trial participants

Patients were enrolled in the trial if they were over 50 years of age with mild to moderate AD according to AD Assessment Scale-cognitive subscale (ADAS-cog; ADAS ≥ 12) and the Clinical Dementia Rating Scale Sum of Boxes (CDR-SOB; CDR ≤ 2). Patients were diagnosed according to the Diagnostic and Statistical Manual of Mental Disorders-5 diagnostic criteria. Patients with a history of stroke, severe brain trauma, and toxic drug exposure were excluded. The main characteristics of the patients are summarised in Additional file 1: Dataset S1. The inclusion, exclusion, and randomisation criteria are described in detail in Additional file 2: Supplementary Appendix.

### Randomisation, interventions, and follow-up

Patients were randomly assigned to receive CMA or placebo (2:1). Patient information (patient number, date of birth, initials) was entered into the web-based randomisation system, and the randomisation codes were entered into the electronic case report form. All clinical staff were blinded to treatment, as were the participants.

Treatment started on the day of diagnosis. Both placebo and CMA were provided in powdered form in identical plastic bottles containing a single dose to be dissolved in water and taken orally, one dose in the morning after breakfast and one dose in the evening after dinner. Each dose of CMA contained 12.35 g *L*-serine (61.75%), 1 g nicotinamide riboside (5%), 2.55 g N-acetyl-*L*-cysteine (12.75%), and 3.73 g *L*-carnitine tartrate (18.65%). All patients received one dose daily after dinner during the first 28 days and two doses after breakfast and dinner, respectively, from day 28 to day 84. All patients came for a follow-up visit on day 84. Further information is provided in the study protocol (Additional file 2: Supplementary Appendix).

### Outcomes

The primary endpoint in the original protocol was to assess the clinical efficacy of CMA in AD patients. For this purpose, cognitive function and daily living activity were assessed by ADAS-Cog, AD Cooperative Study—Activities of Daily Living (ADCS-ADL) and Mini-Mental State Examination (MMSE) after 12-week administration, and compared between the placebo and the treatment arms. The secondary aim of this study was to evaluate the safety and tolerability of CMA. All protocol amendments were authorised and approved by the sponsor, the institutional review board, the independent ethics committee, and the pertinent regulatory authorities. Sample size was estimated by statistical power analysis (Additional file 2: Supplementary Appendix).

The number and characteristics of adverse events, serious adverse events, and treatment discontinuation due to CMA were reported as key safety endpoints from the beginning of the study to the end of the follow-up period. The changes in vital signs, baseline values, and treatment status were recorded on days 0 and 84. A complete list of the endpoints is provided in Additional file 2: Supplementary Appendix.

### Proteomics analysis

Plasma levels of proteins were determined with the Olink panels (Olink Bioscience, Uppsala, Sweden). Briefly, each sample was incubated with DNA-labelled antibody pairs (proximity probes). When an antibody pair binds to its corresponding antigens, the corresponding DNA tails form an amplicon by proximity extension, which can be quantified by high-throughput, real-time PCR. Probe solution (3 μl) was mixed with 1 μl of sample and incubated overnight at 4 °C. Then 96 μl of extension solution containing extension enzyme and PCR reagents for the pre-amplification step was added. The extension products were mixed with detection reagents and primers and loaded on the chip for qPCR analysis with the BioMark HD System (Fluidigm Corporation, South San Francisco, CA). To minimise inter- and intra-run variation, the data were normalised to both internal and interplate control. Normalised data were expressed in arbitrary units (Normalized Protein eXpression, NPX) on a log2 scale and linearised with the formula 2NPX. A high NPX indicates a high protein concentration. The limit of detection, determined for each of the assays, was defined as three standard deviations above the negative control (background).

### Untargeted metabolomics analysis

Plasma samples were collected on days 0 and 84 for untargeted metabolite profiling by Metabolon (Durham, NC). The samples were prepared with an automated system (MicroLab STAR, Hamilton Company, Reno, NV). For quality control purposes, a recovery standard was added before the first step of the extraction. To remove proteins and dissociated small molecules bound to protein or trapped in the precipitated protein matrix and recover chemically diverse metabolites, proteins were precipitated with methanol under vigorous shaking for 2 min and centrifuged. The resulting extract was divided into four fractions: one each for analysis by ultraperformance liquid chromatography-tandem mass spectroscopy (UPLC-MS/MS) with positive ion-mode electrospray ionisation, UPLC-MS/MS with negative ion-mode electrospray ionisation, and gas chromatography-mass spectrometry; and one fraction was reserved as a backup.

### Determination of clinical variables informing response to CMA administration

The patient groups with low and high levels of each clinical parameter were established based on the median score for that clinical parameter across all patients on day 0. Patients scoring at or below the median were placed in the low group; patients scoring above the median were placed in the high group. ADAS-Cog scores were measured over different time points, and statistical significance was tested between time points by using a paired *t*-test. Clinical parameters were deemed informative for the response to CMA if precisely one group (low or high) exhibited more statistically significant changes in ADAS-Cog in the CMA group than in the placebo group.

### Magnetic resonance imaging (MRI) parameters and analysis

Among the entire patient cohort, 40 MRI-compatible patients, 29 in the CMA group and 11 in the placebo group, were recruited for the structural MRI study. Structural MRI was recorded in the 1.5 T SIGNA Explorer MRI device with a 16-channel head coil (General Electric Company, USA).

Hippocampal subfield segmentation and grey/white matter volumetric segmentation were performed using FreeSurfer image analysis software (version 6.0.0) and the integrated hippocampal subfield segmentation module [41]. All T1-weighted images were preprocessed with the standard Freesurfer processing pipeline using the “recon-all” script. In addition to the default processing pipeline, the high-resolution T2-weighted images of each participant were submitted using the “hippocampal-subfield-T1” measure. The Freesurfer algorithm segments 12 hippocampal subfields: hippocampal tail, subiculum, CA1, hippocampal fissure, presubiculum, parasubiculum, molecular layer, granule cell layer of the DG, CA2–3, CA4, fimbria, and the hippocampal–amygdaloid transition area. The volume estimates of these subfields (combined for the right and left hemispheres of each subfield) were then used in the final analysis.

### Image processing

To obtain hippocampal subfield measurements, each T1 image was processed using FreeSurfer version 7.1.0 (http://surfer.nmr.mgh.harvard.edu/). A standard and automatic reconstruction algorithm was used for pre-processing [42, 43] and hippocampal subfield segmentation steps [41]. The hippocampal subfield segmentation of each subject was visually inspected and determined to be free from errors by two independent researchers. Outliers of each subregion volume were defined as data more than 1.5 interquartile range below the first quartile or above the third quartile, and these data were included in the analyses. We used the composite subfield definitions based on the detailed segmentations performed by FreeSurfer [44] and defined the anterior region as the sum of the regions CA1, CA3, CA4, molecular layer, granular cell layers of the dentate gyrus (GC/DG), subiculum, and presubiculum in the hippocampal head. The posterior region consisted of the same subregions in the hippocampal body and the hippocampal tail. The CA composite region was defined as the sum of the volumes of the CA1 region, CA3 region, subiculum, and molecular layer. The DG composite region included the CA4 region and the GC/DG. The subiculum composite region was defined as the presubiculum of the FreeSurfer subfields. The volume of each subfield was calculated separately in the anterior (head) and posterior (body) regions of the hippocampus and hippocampal brain volume was standardised by dividing each by the intracranial volume (ICV), giving ICV-corrected regional brain volume data.

Cortical thickness was measured using the FreeSurfer image analysis suite (V6.0.0, http://surfer.nmr.mgh.harvard.edu/) by computing the averaged distance between the grey/white matter boundary and pial surface at each vertex on the cortical surface. Longitudinal analysis was performed with FreeSurfer image analysis and FreeSurfer's longitudinal processing pipeline program (V6.0.0, http://surfer.nmr.mgh.harvard.edu/) for unbiased subject-specific T1 MRI scans for each subject [45–47]. After several imaging processing steps (skull stripping, Talairach transformation, atlas registration, spherical surface maps and parcellations) based on the subject-specific templates, the cerebral cortex was parcellated into 68 distinct anatomical regions, the averaged thickness was determined, and each subject-specific map was visually analysed before further analysing steps [47].

### Statistical analysis

Paired *t*-test was used to identify the differences in clinical parameters between time points, and one-way ANOVA was used to find the shifts between CMA and placebo groups at each time point. Cohen’s d effect size was estimated by R package “effsize” and paired parameter (paired = TRUE) was used when we compared difference between visits. For the analysis of plasma metabolomics, we removed the metabolite profiles with more than 50% missing values across all samples. Metabolite changes between time points were analysed by paired *t*-test. Metabolite changes between CMA and placebo groups were analysed by one-way ANOVA. Missing values were removed in pairwise comparison. The *P* values were adjusted by Benjamini & Hochberg method. Metabolites with a false-discovery rate of 5% were considered statistically significant.

For analysis of plasma proteomics, we removed the protein profiles with more than 50% missing values across all samples. A paired *t*-test was used to identify the changes between time points, and one-way ANOVA was used to determine the changes between different groups.* P* < 0.01 was considered statistically significant. Spearman correlation analysis was used to analyse the association between CMA and clinical parameters or metabolomics or proteomics.

For structural MRI analysis, we performed a Wilcoxon test on the hippocampal volume change post- vs pre-administration to show the treatment effect. We carried out a comprehensive analysis, focusing on identifying significant interactions in the following areas: pre- and post-treatment group differences and time effects (pre- and post-treatment differences adjusted for active and placebo groups) as previously defined by Clarkson et al. 2018 [48]. By using longitudinal analysis paradigm, we created GLM design matrix for active and placebo groups consisting of (a) CMA and placebo patients at day 0 and day 84 images, (b) baseline of day 0 and day 84 combined image and (c) time difference (year). Similarly, for cortical thickness analysis, the symmetrised percent change (spc) was used as the rate concerning the average thickness: spc = rate / avg [46]. We used positive and negative Monte Carlo simulation with the threshold of 1.3 to define areas with significantly changed thickness (*P* < 0.05).

### Generation of multi-omics network

A multi-omics correlation network was generated based on all patients' clinical parameters, serum chemistry, metabolomics, and proteomics data, following the multi-omics network generation pipeline from iNetModels [49]. Spearman correlations between analytes were calculated using the SciPy package in Python 3.7. Missing values were removed pairwisely by setting the “nan_policy” variable to “omit”. Significant correlations (FDR < 5%) were kept and used to link analytes from the same and different omics. The downstream analysis, i.e., centrality analysis, was performed using the degree centrality calculation in iGraph Python package in Python 3.7.

## RESULTS

### CMA improves cognition and clinical parameters in AD patients

**Fig. 1:**
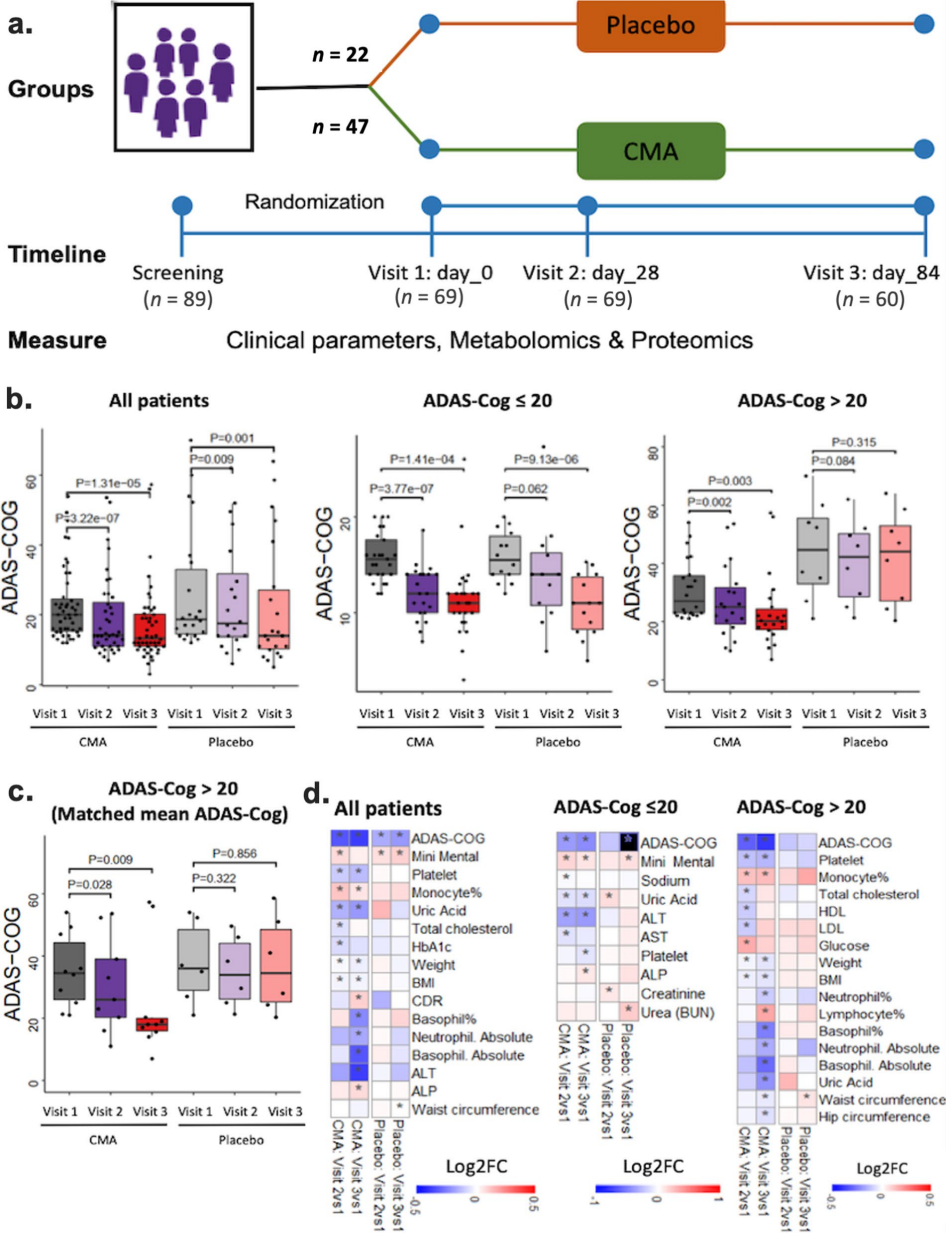
CMA improves ADAS-Cog scores and clinical parameters. **a** Study design for testing the effects of CMA in AD patients. **b** Differences in ADAS-Cog scores in the CMA and placebo groups on days 0, 28 and 84. The ADAS-Cog scores were further analysed by stratifying the patients into high- (score > 20) and low-ADAS-Cog score (≤ 20) groups. The ADAS-Cog score was significantly decreased on day 28 vs day 0 (Log2FoldChange [FC] =  − 0.33, 26% improvement, *P* = 0.0000003) and on day 84 vs day 0 (Log2FC =  − 0.37, 29% improvement, *P* = 0.00001) in the CMA group. A slight but significant decrease was found in the placebo group on day 28 vs day 0 (Log2FC =  − 0.16, 12% improvement, *P* = 0.009) and on day 84 vs day 0 (Log2FC = −0.19, 14% improvement, *P* = 0.001). In addition, the ADAS-Cog score was significantly decreased on day 28 vs day 0 (Log2FC =  − 0.31, 24% improvement, *P* = 0.002) and on day 84 vs day 0 (Log2FC =  − 0.38, 30% improvement, *P* = 0.003) in the high-score CMA group, while no significance difference was seen in the high-score placebo group. **c** We selected 10 patients from the severe (ADAS-COG score > 20) CMA group with matched ADAS-COG values to the placebo group (*P*-value: 0.693) and presented the ADAS-Cog scores. We recalculated the differences in ADAS-COG scores and found significant improvement in the CMA group whereas there was no significant difference in the placebo group. **d** Heatmaps showing log2FC-based alterations of the clinical variables before vs after treatment in both CMA and placebo groups. Asterisks indicate statistical significance based on Student’s *t*-test (*P* < 0.05)

To test the effect of CMA in AD patients, we performed a double-blinded, randomised, placebo-controlled phase 2 study and screened 89 adults diagnosed with AD. We recruited 69 patients older than 50 years with mild to moderate AD according to ADAS-Cog (ADAS ≥ 12) and CDR-SOB (CDR ≤ 2) between February 1 and October 1, 2020. Of the 69 patients, 47 were randomly assigned to the CMA group and 22 to the placebo group, and they completed visit 2 after 28 days. Nine patients (7 in CMA, 2 in placebo groups) dropped out from the study before the day-84 visit due to the COVID-19 lockdown. At the end, 60 patients (40 patients in the CMA group and 20 patients in the placebo group) completed visit 3 on day 84 (Fig. 1a, Additional file 3: Fig. S1). We assessed the clinical variables on days 0, 28 and 84, and analysed the differences between the CMA and placebo groups (Additional file 1: Dataset S1 and Additional file 4: Dataset S2).

The patients' mean age in the study was 70.8 years (56–86 years), and 52.1% were men (Table 1, Additional file 1: Dataset S1). The mean ADAS-Cog score was 22.88 (± 10.51) for CMA and 26.28 (± 17.35) for placebo (Table 1, Additional file 4: Dataset S2). There was no significant difference at the baseline in these demographic parameters and AD clinical indicators between the CMA and placebo groups (Table 1). The other clinical characteristics were similar between the CMA and placebo groups (Additional file 1: Dataset S1 and Additional file 4: Dataset S2). Regarding safety, no severe adverse events occurred, and 5 of the patients (7.2%) reported adverse events. All decided to complete the study (Table 2).

**Table 1:** Demographics and baseline characteristics of the study population*

	CMA (*n* = 47)	Placebo (*n* = 22)	*P* value
Age (years)	70.77 ± 8	70.91 ± 7.54	0.97
Gender			
Male	25 (53.2%)	11 (50%)	0.88
Female	22 (46.8%)	11 (50%)	
Ethnicity	Caucasian (100%)	Caucasian (100%)	–
Body Mass Index	29.04 ± 5.25	27.43 ± 5.05	0.23
ADCS-ADL	55.79 ± 15.02	56.67 ± 15.63	0.83
ADAS-Cog	22.88 ± 10.51	26.28 ± 17.35	0.32
MMSE	19.45 ± 4.21	17.95 ± 5.66	0.22
CDR	0.82 ± 0.45	1.02 ± 0.6	0.09

^*^ Presented as Mean ± Standard deviation, except for gender and ethnicity

*MMSE* Mini Mental state examination, *ADAS-cog* Alzheimer's disease assessment scale-cognitive subscale, *ADCS-AD* Alzheimer's disease cooperative study—activities of daily Living, *CDR* Clinical dementia rating scale

**Table 2:** List of adverse effects

Patient No	Treatment	Adverse event	System organ class	Adverse effect intensity	Relationship to investigational medicinal product
TR10006	Active	Pruritus	Skin and subcutaneous tissue disorders	Mild	Unrelated
TR10011	Active	Dizziness	Nervous system disorders	Mild	Unrelated
TR10034	Active	Diarrhea	Gastrointestinal disorders	Moderate	Unrelated
TR10036	Active	Diarrhea	Gastrointestinal disorders	Moderate	Unrelated
TR20045	Active	Nausea	Gastrointestinal disorders	Moderate	Unknown

We measured clinical variables in all patients and analysed the differences before and after administration in the active and placebo groups (Fig. 1b, Table 3, Additional file 4: Dataset S2). The ADAS-Cog score was significantly decreased on day 28 vs day 0 (Log2FoldChange [FC] =  − 0.33, 26% improvement, *P* = 0.0000003, effect size =  − 0.43, 95% CI = [− 0.59, − 0.29]) and further decreased on day 84 vs day 0 (Log2FC =  − 0.37, 29% improvement, *P* = 0.00001, effect size =  − 0.50, 95% CI = [− 0.71, − 0.29]) in the CMA group, indicating improved cognitive function in AD patients. A slight but significant improvement was also found in the placebo group on day 28 vs day 0 (Log2FC =  − 0.16, 12% improvement, *P* = 0.009, effect size =  − 0.16, 95% CI = [− 0.27, − 0.05]) and day 84 vs day 0 (Log2FC =  − 0.19, 14% improvement, *P* = 0.001, effect size =  − 0.23, 95% CI = [− 0.35, − 0.11]). There was no significant difference between groups on days 28 and 84. This could be related to the placebo effect, which is apparent in early stages of AD clinical trials showing relatively unchanged response or even improvement that may continue for up to 12 months, followed by natural disease progression overtime when the drug effect becomes more prominent while the placebo response decreases [50].

**Table 3:** Differences in ADAS-Cog, ADCS-ADL and MMSE scores in the CMA and placebo groups

Measurements	Patients	Placebo				CMA				CMA vs Placebo					
		Log2FoldChange		*P* value		Log2FoldChange		*P* value		Log2FoldChange			*P* value		
		Day 28 vs 0	Day 84 vs 0	Day 28 vs 0	Day 84 vs 0	Day 28 vs 0	Day 84 vs 0	Day 28 vs 0	Day 84 vs 0	Day 0	Day 28	Day 84	Day 0	Day 28	Day 84
**ADAS-COG**	**All**	− 0.159	− 0.191	**0.009**	**0.001**	− 0.327	− 0.371	**3.2e-07**	**1.3e-05**	− 0.200	− 0.368	− 0.379	0.317	0.111	0.179
	**High score**	− 0.106	− 0.074	0.084	0.315	− 0.305	− 0.381	**0.002**	**0.003**	− 0.522	− 0.671	− 0.774	**0.013**	**0.008**	**0.007**
	**Low score**	− 0.213	− 0.596	0.062	**9.1e-06**	− 0.387	− 0.449	**3.7e-07**	**1.4e-04**	− 0.011	− 0.218	0.143	0.886	0.119	0.478
	**MRI**	− 0.131	− 0.233	0.170	**0.022**	− 0.262	− 0.391	**5.9e-05**	**3.3e-04**	− 0.388	− 0.520	− 0.546	0.159	0.100	0.135
**ADCS-ADL**	**All**	− 0.024	− 0.036	1.000	0.332	0.062	0.002	0.104	0.745	− 0.022	0.063	0.015	0.830	0.557	0.899
**Mini Mental**	**All**	0.078	0.108	**0.043**	**0.022**	0.087	0.048	**0.015**	0.120	0.115	0.124	0.055	0.225	0.249	0.627

*MMSE* Mini mental state examination, *ADAS-cog* Alzheimer's disease assessment scale-cognitive subscale, *ADCS-AD* Alzheimer's disease cooperative study—activities of daily living, *ADAS-Cog score* > 20 is high, ≤ 20 is low; *MRI* Magnetic resonance imaging. Bold indicates statistical significance (*P* < 0.05)

We also analysed the differences in clinical parameters by stratifying the patients into low-score (ADAS-Cog score ≤ 20, *n* = 39) and high-score (ADAS-Cog score > 20, *n* = 30) ADAS-Cog groups. We found a significant difference (Log2FC =  − 0.77, *P* = 0.0073, effect size =  − 1.23, 95% CI = [− 0.3, − 2.17]) between the CMA and placebo groups in patients with higher ADAS-Cog scores on day 84 (Fig. 1b, Additional file 4: Dataset S2). Moreover, we found a significant improvement of ADAS-Cog score on day 28 vs day 0 (Log2FC =  − 0.31, 24% improvement, *P* = 0.002, effect size =  − 0.48, 95% CI = [− 0.76, − 0.2]) and on day 84 vs day 0 (Log2FC =  − 0.38, 30% improvement, *P* = 0.003, effect size =  − 0.59, 95% CI = [− 0.97, − 0.21]) in the high-score CMA group and no significant difference in the high-score placebo group at both time points (Fig. 1b, Additional file 4: Dataset S2). We observed a significant difference in the baseline value distribution and mean of ADAS-Cog scores between the severe (ADAS-Cog > 20) CMA and placebo groups (Fig. 1b) due to the randomisation of the subjects. To verify our results, we randomly selected 10 patients from the CMA group with matched ADAS-Cog values to the placebo group (*P*-value: 0.693) (Fig. 1c), and found significant improvement in the CMA group but no significant difference in the placebo group. Our results indicated that the severe AD patients with high ADAS-Cog scores are more responsive to CMA administration.

Other primary endpoints were ADCS-ADL and MMSE. No significant difference was found between time points or between groups in ADCS-ADL (Fig. 1b, Table 3, Additional file 4: Dataset S2). For MMSE, a significant difference was seen in the placebo group for comparison of day 28 and day 84 vs day 0 (*P* = 0.04 and 0.02, respectively) as well as in the CMA group when comparing day 28 to day 0 (*P* = 0.02) but not for day 84 vs day 0 (Fig. 1b, Table 3, Additional file 4: Dataset S2). There was no significance in ADCS-ADL and MMSE at any time points or between groups in patients evaluated with MRI (Additional file 4: Dataset S2).

Analysis of secondary outcome variables showed that serum alanine aminotransferase (ALT) (Log2FC =  − 0.38, *P* = 0.01) and uric acid levels (Log2FC =  − 0.19, *P* = 0.001) were significantly lower on day 84 vs day 0 only in the CMA group (Fig. 1d, Additional file 4: Dataset S2). This reduction was seen both in high- and low-ADAS-Cog score groups. In contrast, we found no significant difference on day 84 vs day 0 in the placebo group (Fig. 1d, Additional file 4: Dataset S2).

We also measured the complete blood count parameters and found that their levels were significantly changed in the CMA group (Fig. 1d, Additional file 4: Dataset S2). We found that the levels of platelets, basophil% and absolute numbers of basophil and neutrophil were significantly lower on day 84 vs day 0 only in the CMA group. In contrast, the levels of monocytes were significantly increased on day 84 vs day 0 in the CMA group (Fig. 1d, Additional file 4: Dataset S2). Hence, our analysis indicated that the administration of CMA improved the clinical parameters in parallel with improved cognitive functions in AD patients.

### Blood profile informs response to CMA

Treatment response variability and clinical heterogeneity are well documented in AD in the literature. Here, we noticed interindividual variability in clinical measures in response to CMA administration. Therefore, we hypothesized that some patients would respond better to CMA than others and that clinical measurements could define these subsets.

**Fig. 2:**
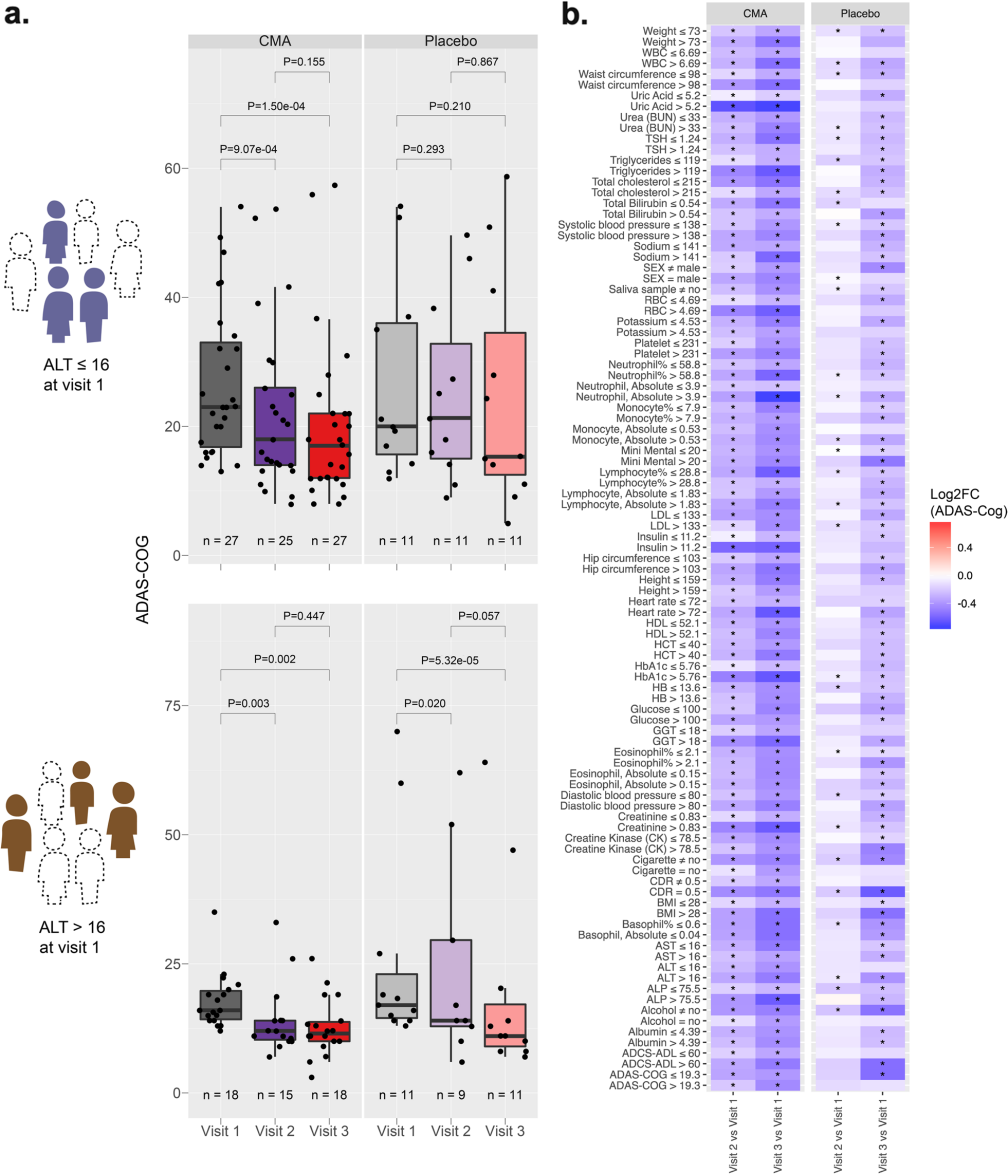
Identification of clinical variables informative for response to CMA administration. **a** Distribution of ADAS-Cog scores over visits for patients with ALT level ≤ 16 IU/l (upper panel) and ALT > 16 IU/l (lower panel) at visit 1. **b** Between-visit changes of ADAS-Cog score in AD patients stratified by other various clinical variables. Only those clinical variable groupings resulted in a more significant change of ADAS-Cog in the CMA group compared to the placebo group is shown (*P* < 0.05). The colour scale indicates log2 fold change of ADAS-Cog score between visits. Statistical significance between visits was determined by a paired *t*-test across individuals who attended both visits. Asterisks indicate a statistical significance of *P* < 0.05

To determine whether ALT, a marker for liver damage, could indicate a better response to CMA, we stratified the patients into high- and low-ALT groups by the median ALT of all patients on day 0. The patients of the CMA group with low ALT levels achieved a significant improvement on ADAS-Cog score over different time points, while the patients in the placebo group had no improvement. In contrast, the patients in the CMA group with high ALT levels also exhibited an improved (i.e., decreased) ADAS-Cog score, but the degree of change was not as much as the low-ALT patients in the CMA group (Fig. 2a). Moreover, patients in the placebo group with high ALT levels also had improved ADAS-Cog scores. Thus, these results suggest that the patients with low ALT levels are more responsive to CMA.

We further repeated stratification for each blood parameter, and identified high alkaline phosphatase (ALP), low gamma-glutamyl transferase (GGT), high hematocrit, high HbA1c, high insulin, high uric acid, high basophil count, and high red blood cell count as indicators for better responsiveness to CMA (Fig. 2b, Additional file 5: Fig. S2).

### CMA increases the plasma levels of metabolites associated with metabolic activators

**Fig. 3:**
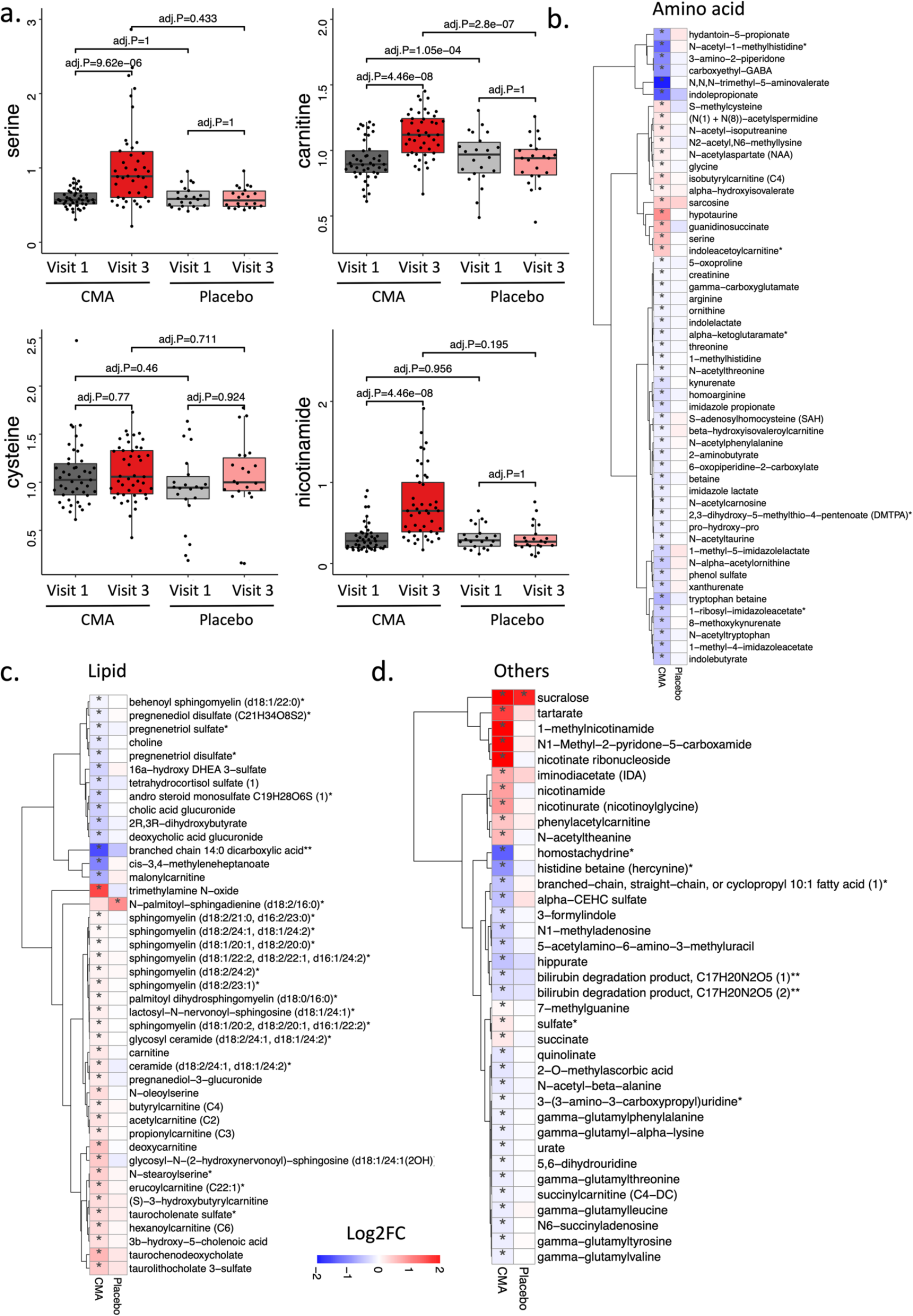
CMA alters plasma metabolite levels. **a** Differences in the plasma levels of individual CMA, including serine, carnitine, cysteine and nicotinamide on days 0 and 84. **b**–**d** Plasma levels of amino acids, lipids and other metabolites that were significantly different between day 84 and day 0 in the CMA and placebo groups. Adjusted *P* < 0.05. Heatmap shows log2FC values of metabolites between day 84 and day 0. Asterisks indicate statistical significance based on paired Student’s *t*-test. Adjusted *P* < 0.05. Log2FC: log2(fold change)

We first analysed the plasma levels of serine, carnitine, NR, and cysteine as well as their by-products. The plasma levels of metabolic activators were increased on day 84 vs day 0 in the CMA group (Fig. 3a, Additional file 6: Dataset S3). Moreover, the plasma levels of NR, 1-methylnicotinamide, nicotinurate, N1-methyl-2-pyridone-5-carboxamide and nicotinamide (associated with NR and NAD+  metabolism); serine, glycine and sarcosine (associated with serine and glycine metabolism); as well as deoxycarnitine and carnitine (associated with carnitine metabolism) in the CMA group were significantly higher on day 84 compared to day 0 (Fig. 3b–d, Additional file 6: Dataset S3).

**Fig. 4:**
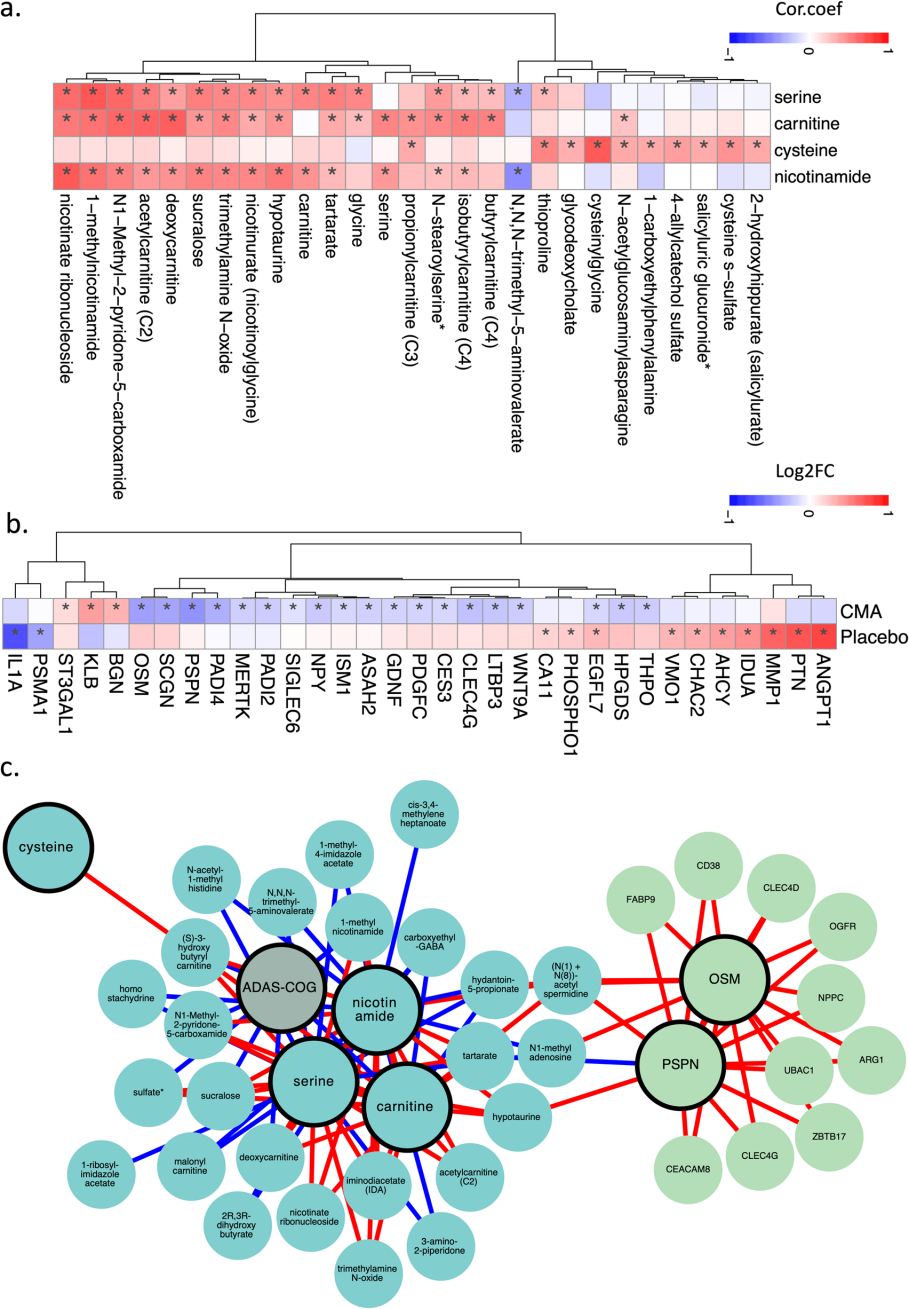
Correlation of CMA with plasma metabolites and altered plasma protein levels. **a** Associations between the plasma levels of individual CMA and the 10 most significantly correlated plasma metabolites. Asterisks indicate statistical significance (Adjusted *P* < 0.05) based on Spearman correlation analysis. Cor.Coeff: Correlation coefficient. **b** Heatmap showing log2FC-based significantly different proteins on day 84 vs day 0 in the CMA and placebo groups. Asterisks indicate statistical significance based on paired Student’s *t*-test (*P* < 0.01). **c** Integrated multi-omics data based on network analysis represent the neighbours of the CMA, including serine, carnitine, nicotinamide and cysteine, and ADAS-Cog scores. Only analytes that were significantly altered in CMA on day 84 vs day 0 are highlighted

Next, we investigated the relationship between the plasma level of administered metabolic activators and other metabolites. We analysed 195 of the plasma metabolites most significantly correlated with serine, *L*-carnitine, NR, and cysteine (Additional file 7: Dataset S4). We found two clusters of metabolites that are significantly correlated with cysteine only or together with serine, carnitine and NR (Fig. 4a). We observed that cysteine had different plasma changes than the other three metabolic activators, as reported in previous clinical trials [38, 51].

### Effect of CMA on global metabolism

We identified the significantly (FDR < 0.05) different plasma metabolites on day 84 vs day 0 and found that the plasma levels of 132 metabolites were significantly different in the CMA group (Fig. 3, Additional file 6: Dataset S3). Evaluation of plasma metabolites that differed significantly on day 84 vs day 0 in each group showed that a larger number of metabolites related to amino acid metabolism (*n* = 53), lipid metabolism (*n* = 42) and other metabolic pathways (*n* = 37) were altered in the CMA group compared to the placebo group (Fig. 3, Additional file 6: Dataset S3).

N-acetyl aspartate (NAA) is one of the most abundant brain metabolites, and its reduced plasma levels are associated with brain tissue damage. Previous research has revealed the importance of NAA in maintaining energy metabolism in the central nervous system [52]. Here, we observed that the plasma levels of NAA significantly increased on day 84 vs day 0 in the CMA group (Fig. 3b, Additional file 6: Dataset S3). Another upregulated metabolite on day 84 vs day 0 in the CMA group was sarcosine (a derivative of glycine), which has been widely studied for its effect to improve cognitive symptoms through different pharmacological activities in neurons [53]. Of note, quinolinic acid (an endogenous excitotoxin acting on *N*-methyl-*D*-aspartate receptors leading to neurotoxic damage) was significantly decreased on day 84 vs day 0 only in the CMA group (Fig. 3b, Additional file 6: Dataset S3).

Increased plasma levels of homocysteine are a known risk factor for AD, and several animal studies have implicated promising effects of methionine restriction [54, 55]. In our clinical trial, plasma levels of *S*-adenosylhomocysteine, 2,3-dihydroxy-5-methylthio-4-pentenoate and *N*-acetyl taurine were significantly downregulated on day 84 vs day 0 in the CMA group (Fig. 3b, Additional file 6: Dataset S3). Of note, reductions of these metabolites were significantly correlated with serine and NR supplementation (Fig. 4a, Additional file 7: Dataset S4).

Increased plasma levels of metabolites in the kynurenine pathway are associated with AD severity [54]. In our study, we found that plasma levels of kynurenate and 8-methoxykynurenate were significantly lower on day 84 vs day 0 in the CMA group (Fig. 3b, Additional file 6: Dataset S3). Reduction of the plasma level of kynurenate was positively correlated with plasma serine levels (Additional file 7: Dataset S4). Kynurenate, which has a prooxidant effect, is the product of the tryptophan degradation pathway. It produces superoxide radicals through aerobic irradiation, which lead to cytochrome *c* reduction [56]. It has been reported that increased levels of kynurenine lead to cell death through the reactive oxygen species pathway in nature killer cells [57] and lower blood pressure in systemic inflammation [58].

Emerging evidence indicates a link between abnormal kidney function and AD, but the potential impact of the kidney on cognitive impairment is still undetermined [59]. Recent studies showed that plasma N,N,N-trimethyl-5-aminovalerate is involved in lysine metabolism, and serves as an indicator of elevated urinary albumin excretion [60]. Here, we found that the plasma level of N,N,N-trimethyl-5-aminovalerate was significantly decreased on day 84 vs day 0 in the CMA group (Fig. 3b, Additional file 6: Dataset S3) and significantly inversely correlated with the plasma levels of serine and NR. Moreover, the plasma level of creatinine was also significantly decreased on day 84 vs day 0 in the CMA group (Fig. 3b, Additional file 6: Dataset S3). The decrease of plasma creatinine inversely correlated with the plasma level of serine (Additional file 7: Dataset S4). Additionally, our analysis revealed decreased levels of several metabolites belonging to histidine metabolism in the CMA group on day 84 vs day 0. Among those, N-acetyl-1-methylhistidine is associated with chronic kidney disease and showed a significant negative correlation with serine supplementation (Fig. 3b, Additional file 7: Dataset S4). Also, plasma levels of metabolites related to the urea cycle (3-amino-2-piperidone, arginine, homoarginine, N-alpha-acetylornithine, ornithine and pro-hydroxy-pro) were significantly decreased in the CMA group on day 84 vs day 0 (Fig. 3b, Additional file 6: Dataset S3) and inversely correlated with the plasma levels of serine and NR (Additional file 7: Dataset S4).

Lipids play a fundamental role in the pathophysiology of neurodegenerative diseases, including AD. Specific lipid species of cellular membranes (e.g., cholesterol and sphingolipids) are structural components of cell membrane and regulate many critical aspects of brain function [61]. In our study, plasma levels of many metabolites associated with sphingomyelins and fatty acid metabolism (acyl carnitines) were significantly increased on day 84 vs day 0 in the CMA group (Fig. 3c, Additional file 6: Dataset S3). Interestingly, plasma levels of pregnenolone steroids and 2R,3R-dihydroxybutyrate were significantly decreased on day 84 vs day 0 (Fig. 3c, Additional file 6: Dataset S3). These alterations were significantly positively correlated with carnitine and serine levels (Additional file 7: Dataset S4).

### Effect of CMA on plasma proteins

Plasma levels of 1466 protein markers were measured with the plasma proteome profiling platform Proximity Extension Assay. After quality control and exclusion of proteins with missing values in more than 50% of samples, 1463 proteins were analysed. Proteins whose levels differed significantly between visits in the CMA and placebo groups are listed in Additional file 8: Dataset S5.

We found that 22 proteins were significantly (*P* < 0.01) different in the CMA group on day 84 vs day 0. Nineteen of these proteins were significantly decreased, whereas 3 were significantly increased on day 84 vs day 0. After filtering out the proteins based on log2FC, we found that the plasma levels of persephin (PSPN), oncostatin-M (OSM), PADI4 (protein-arginine deiminase type-4), PDGFC (platelet-derived growth factor C), SCGN (secretagogin), LTBP3 (latent-transforming growth factor beta-binding protein 3), CLEC4G (C-type lectin domain family 4 member G), Mer tyrosine kinase (MertK), WNT9A (protein Wnt-9a), isthmin-1, ASAH2 (neutral ceramidase), CES3 (carboxylesterase-3), HPGDS (hematopoietic prostaglandin D synthase), pro-neuropeptide Y, thrombopoietin, SIGLEC6 (sialic acid-binding Ig-like lectin-6), GDNF (glial cell line-derived neurotrophic factor), PADI2 (protein-arginine deiminase type-2) and epidermal growth factor-like protein 7 (EGFL7) were significantly downregulated, while KLB (beta-klotho), BGN (biglycan), and CMP-N-acetylneuraminate-beta-galactosamide-alpha-2,3-sialyltransferase-1 (ST3GAL1) were significantly upregulated on day 84 vs day 0 in the CMA group (Fig. 4b, Additional file 8: Dataset S5). Only one protein was significantly (*P* < 0.01) altered in the placebo group (EGFL7, upregulated).

### Integrative multi-omics analysis

Multi-omics data integration can provide novel insights and a more holistic view of the human body in both health and disease states [62]. In this study, we generated an integrative multi-omics network using metabolomics and proteomics data, coupled with detailed clinical variables, to understand the functional relationships between analytes from the same and different omics data types. We generated the network using the method used in iNetModels [49], to which we also deposited our network. The network consists of 937,282 edges from 2273 nodes (36.3% network density, Additional file 9: Dataset S6).

We extracted a sub-network to highlight the interactions between the individual metabolic activators, cognitive function (ADAS-Cog scores), two highlighted proteins (OSM and PSPN), and their top neighbours (Fig. 4c). From the sub-network, ADAS-Cog was negatively associated with carnitine (and its derivatives) and nicotinamide-associated metabolites, whereas the metabolic activators were negatively associated with fatty acid and histidine metabolism. Finally, OSM and PSPN were positively associated with immune- and cell cycle-related proteins.

Subsequently, we performed centrality analysis to identify the most central analytes in the networks. The top 20 most central metabolites were dominated by amino acid metabolites (tryptophan, glutamate, and branched-chain amino acid metabolism) and lipid metabolites (androgenic steroid pathway), and the top proteins were related to, among others, short- and long-term memory (calbindin), lipid metabolism (PLA2G10 [group 10 secretory phospholipase-A2]), and immune response (SELPLG [selectin-P glycoprotein ligand 1], CLEC4D [C-type lectin domain family 4 member E], and galectin-7).

Furthermore, we performed community analysis within the network using the Leiden algorithm. We discovered 3 modules that showed significant interaction among the members. In cluster-0, the biggest cluster, the top nodes were related to tryptophan metabolism (indole acetate), fatty acid metabolism (3-hydroxyoctanoate), and steroid metabolism (11-ketoetiocholanolone glucuronide and 11-beta-hydroxyetiocholanolone glucuronide). Moreover, we found 2 top proteins in the same cluster, alpha-actin-2 and insulin-like growth factor-binding protein 1, associated with AD [63, 64]. In cluster-1, the top nodes were associated with leucine metabolism (3-hydroxy-2-ethylpropionate), ceramide phosphatidylethanolamine, and a carnitine metabolite (erucoylcarnitine); meanwhile, the central nodes of cluster-2 were related to methionine metabolism and aminosugar metabolism (*N*-acetylglucosamine/*N*-acetylgalactosamine). These results showed that the integrative multi-omics network analysis could be used to strengthen the results from single omics analyses and identify key analytes associated with AD. Moreover, it provided new insights by elucidating the functional relationships within and between different omics data.

In evaluating the correlations between each activators (used in the present study for therapeutic purposes) and clinical, metabolic, and proteomic parameters, we identified significant correlations of serine, carnitine, cysteine, and nicotinamide levels with improved peripheral blood parameters, such as liver function, complete blood count (CBC), and glycated hemoglobin (HbA1c), which are relevant to the pathogenesis of AD. Accordingly, improved ADAS-Cog scores were also associated with serum serine and carnitine changes, which fit well with their well-known pro-cognitive and energy-boosting effects. Similar results were also observed for metabolomic and proteomic data. The majority of the activators exhibited significant correlations with improved metabolites and proteins (either increased or decreased) relative to a slower degeneration process in AD. It is worth mentioning here that two of the proteins, OSM and PSPN, most strongly associated with other beneficial protein metabolites, were also related to critical alterations of several amino acids, such as spermidine and hypotaurine, which may suggest a metabolic shift from the protein to the amino acid metabolism to compensate for the energy deficit reported in AD.

### Effect of CMA on hippocampal volumes and cortical thickness

**Fig. 5:**
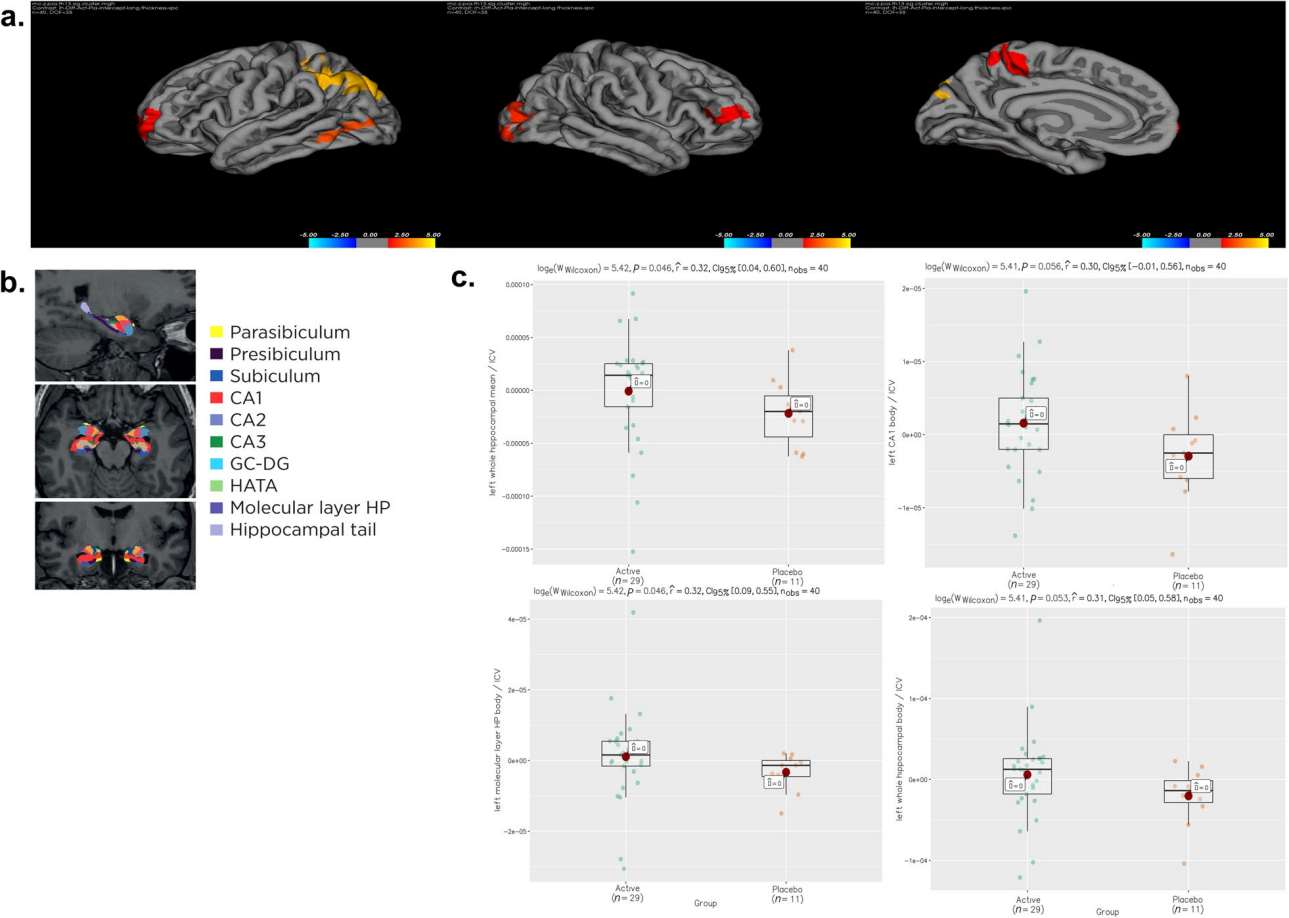
Structural magnetic resonance imaging analysis. **a** Increased cortical thickness (red-yellow) in the study group (*P* < 0.05) in the inferior parietal, lateral occipital and middle frontal and paracentral cortical regions based on the illustration of FreeSurfer's Qdec application. **b** Segmentation maps of the hippocampal subfields displayed on the sagittal (top), axial (middle), and coronal (bottom) planes. **c** Hippocampal subfield measurements showing increased volumes in the study group (*P* < 0.05) in the left mean hippocampal (upper left) and left hippocampal molecular layer (lower left) based on the FreeSurfer's Qdec application. Other sub-anatomic hippocampal regions iii) including left CA1 body (upper right) and iv) left whole hippocampal body (lower right) were not significant but near to a statistically significant level (*P* > 0.05)

The baseline demographics and improved clinical parameters of the MRI group significantly aligned with the entire patient cohort (Additional file 1: Dataset S1, Additional file 4: Dataset S2). According to the longitudinal cortical thickness analysis, the active group showed statistically significant alterations in the bilateral occipital, bilateral rostral middle frontal, left inferior parietal, and left paracentral regions compared to the placebo group (Fig. 5a). Additionally, we evaluated the sub-anatomic hippocampal regions shown in Fig. 5b. By comparing the differences between pre- and post-treatment groups in each treatment arm, our results showed that the left whole hippocampal mean volume (expressed as the average volume of the hippocampal body and the hippocampal head) and the volume of left molecular layer of the hippocampal body were maintained after the CMA treatment; however, they were significantly reduced in the placebo group (*P* < 0.05, Fig. 5c, Additional file 10: Dataset S7). Other sub-anatomic hippocampal regions (left CA1 body and left whole hippocampal body) were not significant between drug and placebo but near a statistically significant level (*P* = 0.055 and *P* = 0.052, respectively, Additional file 10: Dataset S7).

## Discussion

Our results suggested that oral administration of CMA for 84 days has a considerable effect on cognitive function in AD patients based on ADAS-Cog scores. The cognitive functions in patients with high ADAS-Cog scores were improved in the CMA group while there were no significant differences in the placebo group. We also showed beneficial effects in both severe and mild patients. Cognitive functions of AD patients were improved by 29% in the CMA group, whereas they were improved only by 14% in the placebo group after 84 days, consistent with a placebo effect that is seen in other AD clinical trials [50, 65, 66]. The improvement of cognitive function was supported by positive alterations in cortical thickness and maintenance of the hippocampal subfield volumes in the CMA group, while no cortical thickness difference but significant volume decline was found in the placebo group. Our finding of a possible beneficial effect in severe AD is of particular value since severe AD patients lack current therapeutic regimes, except for palliative support. Apart from clinical severity, we observed that various clinical variables were also related to the treatment response. For example, patients with low ALT and increased metabolic load (i.e., increased HbA1c and insulin levels) or impaired CBC values responded better to CMA treatment.

The effect of oral administration of CMA was substantiated with a comprehensive analysis of proteins and metabolites in the plasma of patients using a multi-omics analytical platform. The clinical results were consistent with the genome-scale metabolic modelling of more than 600 AD patients showing clear evidence of mitochondrial dysfunction [40]. It is also consistent with the results from an animal model demonstrating improvement in AD-associated histological parameters in animals treated with oral administration of CMA [67]. Thus, the present study suggests a promising therapeutic regime that might help to improve metabolic alterations in AD patients.

Considering the role of hippocampal and frontoparietal degeneration in AD pathogenesis, our neuroimaging observation of improved hippocampal subfield volumes and cortical thickness after metabolic stimulation was not surprising. Herein, we observed significantly improved hippocampal volumes as well as frontal and parietal cortical thickness in the CMA group, whereas no positive effects were observed in the placebo group. Our results suggested a treatment effect in the CMA group in major cognitive brain regions in AD.

Numerous studies in healthy participants have suggested that metabolism and cognitive brain network organization are strongly linked [68, 69]. This is especially true for higher-order cognitive tasks, which are the most metabolically demanding for the brain, and "burn out" would make the brain prone to neurodegenerative and age-related alterations marked by neuronal metabolic dysfunction [70]. Metabolic cofactors improve mitochondrial metabolism by a three-step strategy: (1) *L*-carnitine to enhance the transport of fatty acids across the mitochondrial membrane, (2) the NAD+  precursor nicotinamide riboside to enhance the β-oxidation of fatty acids in mitochondria, and (3) GSH precursors including serine and NAC to form GSH that is required to protect liver against oxidative stress mediated by free radicals, which are generated through increased β-oxidation of fatty acids in the mitochondria. Thus, our results of the pro-cognitive benefits of CMA may be connected to the metabolic load of cognitive networks and their responsiveness to such metabolic interventions.

Growing data indicate that significant mitochondrial dysfunction occurs in AD pathogenesis [71]. In our previous studies, we reported that CMA administration effectively facilitates fatty acid oxidation in the mitochondria and alleviates oxidative stress by de novo GSH synthesis [34–39]. However, how these beneficial effects contribute to both structural and cognitive abnormalities is unknown and needs further evaluation. Based on the current literature, enhancing NAD+  levels may help restore brain energy metabolism and oxidative stress, which are implicated in cognitive decline. Studies in animal models have shown that NAD+  precursors such as NR and NMN can normalize neuroinflammation, improve learning and motor functions, induce mitophagy, and protect against neurological damage [72–77]. In parallel, preserving the redox homeostasis in elderly animals has been shown to protect against cognitive decline, potentially through preserving NMDA receptor activation [78]. A recent study reported that astrocytic glycolysis controls cognitive functions through synaptic NMDA receptors and suggested oral *L*-serine as an accessible therapy for AD patients [79]. Similarly, NAC therapy has been shown to restore NMDA receptor activation and reduce oxidative injury in the hippocampus of PD animal models [80]. While removal of oxidative stress with CMA administration suggests a reasonable biological explanation for the brain hypofunction that accompanies AD-related pathologies, further mechanistic studies should be performed.

The proteomic analysis in this study revealed significant alterations of levels of several critical proteins that play an essential role in the pathogenesis of AD. For instance, levels of MertK [81, 82], EGFR [83, 84], oncostatin [85–89], PAD4 [90, 91], LTGF [92–96], and TPO [97], known as a potent inducer of neuro-inflammation, amyloid production and apoptosis, were significantly decreased. In contrast, proteins with neuroprotective and pro-cognitive properties, such as Klotho [98, 99] and ST3GAL1 [100], werer increased after CMA treatment. More interestingly, most of the analysed proteins were also shown to be significantly altered in recent human AD studies [92–96, 101–107]. KlothoB levels were also significantly altered after CMA treatment, consistent with its neuroprotective role as a cofactor and neurotrophic factor. Recent studies have shown that KlothoB indirectly regulates glucose and energy metabolism through F2F1, which is expressed in some regions of the brain and involved in learning and memory [106]. Moreover, gamma-aminobutyric acid (GABA) signalling has also been shown to play a critical role in mediating the detrimental effects of increased dihydroxybutyrate in the progression of mild cognitive impairment (MCI) [108]. Interestingly, we found that the dihydroxybutyrate levels were decreased after treatment based on metabolomic analysis. Although the exact pathways involved in the metabolic generation of DHBA are still far from clear, it has been hypothesized that dihydroxybutyrate levels may be a compensatory response to increased cellular stress secondary to compromise of the Krebs cycle function, creating an alternative energy production pathway in AD [108]. This represents indirect evidence for the energetic regulatory effect of CMA. Our findings are also in agreement with a recent study by Johnson et al., which evaluated > 2000 brains and nearly 400 cerebrospinal fluid samples by quantitative proteomics and identified mitochondrial metabolism as one of the most affected six metabolic pathways showing a strong correlation with the overall cognitive and pathological changes in AD [109]. Hence, we observed that the significantly improved proteins related to mitochondrial bioenergetic mechanisms and the neuroinflammatory process were associated with increased cognitive scores and preserved hippocampal volumes after CMA treatment.

The metabolomics data were consistent with the expected biological outcomes of CMA treatment. Levels of plasma nicotinamide and related metabolites were increased, suggesting that NR provides sufficient substrate for mitochondrial fatty acid oxidation. In addition to its role as a cellular metabolite, NAD+  functions as an essential cofactor for the DNA repair protein poly (ADP ribose) polymerase 1 (PARP1) [110]. Hyperactivation of PARP1 and decreased NAD+  have already been identified in the brains of patients with AD [111, 112]. Plasma levels of serine were also increased, suggesting that CMA treatment improves the serine deficiency associated with AD. For instance, a recent study showed that the adenosine triphosphate (ATP)-reducing effect of glucose hypometabolism is restored with oral serine supplementation, suggesting the potential use of oral serine as a ready-to-use therapy for AD [79]. The exact mechanism of action also applies to cysteine. As a GSH precursor, cysteine acts as an antioxidant and anti-inflammatory agent, maintaining the mitochondrial homeostasis and key neurotransmitter systems, such as glutamate, involved in learning and memory [113, 114]. Accordingly, NAC has been tested for treatment of AD and shown potentials for use as an alternative medication [115]. More importantly, fatty acid oxidation and carnitine metabolism were significantly facilitated, as shown by the robust increase in plasma levels of carnitine. These findings fit well with recent human data showing that severe disturbances in carnitine metabolism frequently occur in individuals with AD, in association with severe mitochondrial dysfunction [116, 117]. Cristofano et al. showed a progressive decrease in carnitine serum levels in individuals shifting from normal status to AD, suggesting that decreased serum concentrations of carnitine may predispose them to AD [118]. In support of this hypothesis, human clinical studies have demonstrated pro-cognitive effects of carnitine in MCI and AD [119–121].

In addition, the levels of tryptophan metabolites, including kynurenate, kynurenine, and tryptophan betaine, decreased significantly after CMA treatment. Increased levels of these metabolites are associated with increased severity of neurodegeneration and clinical cognitive impairment through a high oxidative load, and with the formation of neurofibrillary tangles (NFTs) [122, 123]. For instance, recent data showed a synergistic relationship between β-amyloid 1–42 and enzymatic activations of the tryptophan kynurenine pathway, resulting in increased oxidative stress, which may be associated with the formation of NFTs and senile plaque development [124]. Also, a recent study revealed that tryptophan-2,3-dioxygenase is highly expressed in the brains of AD patients and co-localised with quinolinic acid, NFTs, and amyloid deposits in the hippocampus of post-mortem brains of AD patients [125].

We also observed significantly increased levels of NAA, sarcosine, methionine, cysteine, and *S*-adenosylmethionine and decreased levels of histidine, tryptophan quinolate, and urea cycle metabolites, which play a critical role in cognitive functions. For instance, increased NAA may provide an additional energy source for intercellular metabolite trafficking during the neurodegenerative process, especially when glucose metabolism is downregulated [52]. Similarly, increased sarcosine levels may boost cognition, as previously shown in patients with schizophrenia, a disease in which oxidative damage and impaired glucose metabolism play key roles [126]. In addition, decreased histidine metabolism and other decreased markers, such as homocysteine and *S*-adenosylhomocysteine found in our treatment group, have been shown to slow the cognitive ageing process when appropriately downregulated [127]. For instance, increased plasma homocysteine levels are a known risk factor for AD, whereas a low-leucine and low-arginine diet yields beneficial effects on cognition [128].

Interestingly, CMA rapidly lowered levels of uric acid and associated metabolites. Uric acid stimulates inflammation either directly or by activating NLRP3 inflammasomes [129]. Although the extent to which uric acid reduction contributed to the regression of cognitive impairment was unclear, the effect may be linked to improved metabolic homeostasis. For instance, a recent clinical study showed increased urea metabolism in AD patients [130]. Accordingly, decreased levels of taurine and urea metabolites are associated with a diminished risk of dementia [131].

To date, a few studies have identified the global changes in metabolites and metabolic pathways in AD [25, 132, 133]. Among these, some studies highlight that alterations in lipid meabolism also play an essential role in the pathophysiology of AD [134]. In terms of lipid metabolism, significant differences in the levels of some compounds have been observed in AD patients. Despite some discrepant trends in cross-sectional studies examining lipids in AD patients [135, 136], the plasma levels of sphingolipids, sphingomyelins [137, 138], acylcarnitines [139] and phosphatidylcholines [140–142] exhibited statistically lower concentrations in patients with AD, even in the preclinical stages of the disease [27]. In addition, a significant correlation among different lipid metabolites, tau and amyloid pathology, brain atrophy and cognitive decline has been observed in an AD patient study [27]. An autopsy study of frontal cortex metabolites showed that impaired glycerophospholipid metabolism is involved in six central metabolic pathways that are altered in AD [143]. In brief, we observed significantly increased levels of lipid metabolites after CMA treatment, including sphingomyelin, carnitine and carnitine-related by-products, which were previously reported to decrease in patients with AD.

Despite insufficient clinical AD data concerning cholesterol metabolites and dicarboxylic acids (DCAs), we observed significantly lower levels of these metabolites after CMA treatment [144]. Levels of pregnanediol, a metabolite of pregnenolone, and DCAs, end-products of β- or omega oxidation, which were observed as decreased in the present study, were previously reported to be lower in the urine of patients with AD [145, 146]. Considering the neurotoxic role of bile acids, along with the oxidative properties of DCAs, the detection of decreased levels of bile acid metabolites and DCA products in the present study is therefore not surprising. Similarly, allopregnanolone has already been reported to have harmful effects on cognitive functions through GABA signalling [147]. Also, increased bile acid levels have been reported in MCI and AD. In contrast, bile acids strongly inhibit the cysteine catabolic pathway in the preclinical period, resulting in depletion of the free cysteine pool and reduced GSH concentrations [148].

The study has limitations. One limitation of the study was the small sample size after classifying the patients into low- and high-ADAS-Cog score groups. Therefore, a clinical trial with a larger sample size is necessary to elucidate the effects of CMA on functional and structural brain alterations. Moreover, in future studies, mitochondrial functions and changes of beta-amyloid 1–42, total-tau, and phosphorylated-tau concentrations after CMA administration should be analysed. Another limitation of the study was the lack of ApoE genotyping. Such evaluation would be informative for the detection of risk variants for AD patients.

## Conclusions

The present phase 2 clinical study suggested that oral administration of CMA improves metabolic alterations in AD patients, and that CMA is safe and well-tolerated, with no major safety concerns identified. The safety profile of metabolic activators in AD patients is consistent with the results of our previous one-day calibration study and phase 2 and 3 clinical trials. Importantly, our findings indicated that CMA has positive effects on cognitive functions and markers of metabolic abnormalities, especially in patients with severe AD. Of note, our results should be interpreted with caution until being confirmed by a randomized, double-blinded and placebo-controlled phase 3 clinical trial.

## Supplementary Information

**Additional file 1: Dataset S1. **Collection of samples of CMA and placebo groups and the measured values of clinical indicators before and after treatment.

**Additional file 2: Appendix.**

**Additional file 3: Fig. S1 **Consort flow diagram. Diagram shows the progress through the phases of the parallel randomisation of drug and placebo groups.

**Additional file 4: Dataset S2. **Statistical analysis of clinical indicators between different visits or groups.

**Additional file 5: Fig. S2 **Interindividual variability in clinical measures in responses to CMA administration.

**Additional file 6: Dataset S3. **Plasma metabolomics data for each patient before and after treatment and statistical analysis of plasma metabolites between different visits or groups.

**Additional file 7: Dataset S4. **The association between the plasma level of the four supplements serine, carnitine, cysteine and nicotinamide riboside with the plasma levels of other metabolites.

**Additional file 8: Dataset S5. **Plasma proteomics data were generated with the Olink cardiometabolic, inflammation, neurology and oncology panels for each patient before and after treatment and statistical analysis of plasma proteins between different visits or groups.

**Additional file 9: Dataset S6. **Multi-Omics Network Data, including edges and nodes information. The network is presented in the iNetModels (http://inetmodels.com).

**Additional file 10: Dataset S7.** Structural magnetic resonance imaging analysis results

## Data Availability

The data supporting the findings of this study are available in Supplementary Material. Raw data are available from the corresponding author, upon reasonable request.

## Abbreviations

AD: Alzheimer’s disease
ADAS-Cog: Alzheimer’s disease assessment scale-cognitive subscale
ADCS-ADL: Alzheimer's Disease cooperative study—activities of daily living
ALT: Alanine transferase
CDR-SOB: Clinical dementia rating scale sum of boxes
CMA: Combined metabolic activators
GGT: Gamma-glutamyl transferase
HbA1c: Glycated hemoglobin
MMSE: Mini mental state examination
NR: Nicotinamide riboside
