# Supplementary figures and images for: Combined metabolic activators improve cognitive functions in Alzheimer’s disease patients: a randomised, double-blinded, placebo-controlled phase-II trial
Source: Transl Neurodegener. 2023 Jan 26;12:4. doi: 10.1186/s40035-023-00336-2 (PMC9879258; doi:10.1186/s40035-023-00336-2)

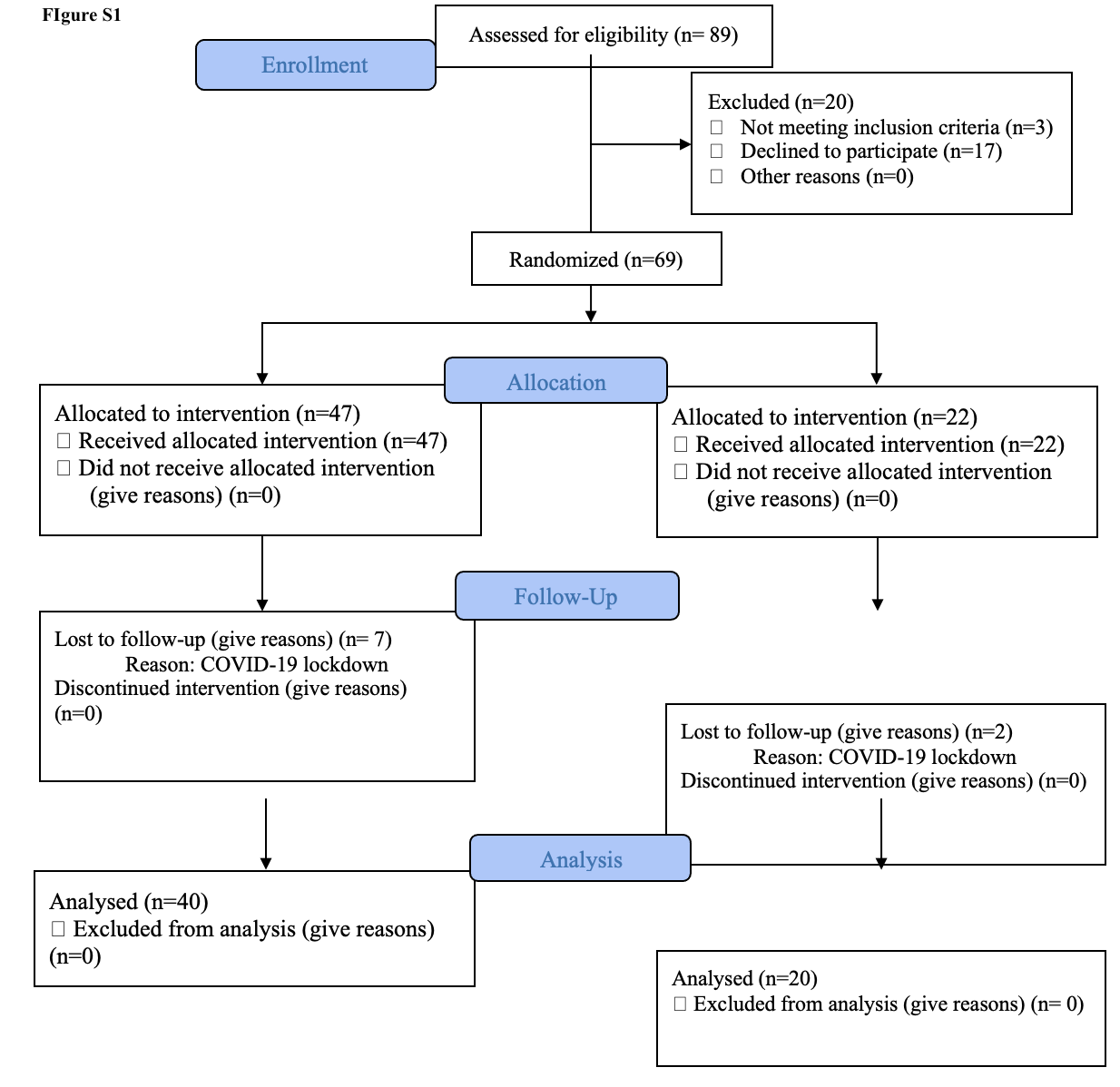

Supplement: Supplementary file 3 — Additional file 3: Fig. S1 Consort flow diagram. Diagram shows the progress through the phases of the parallel randomisation of drug and placebo groups. [file 40035_2023_336_MOESM3_ESM.png]

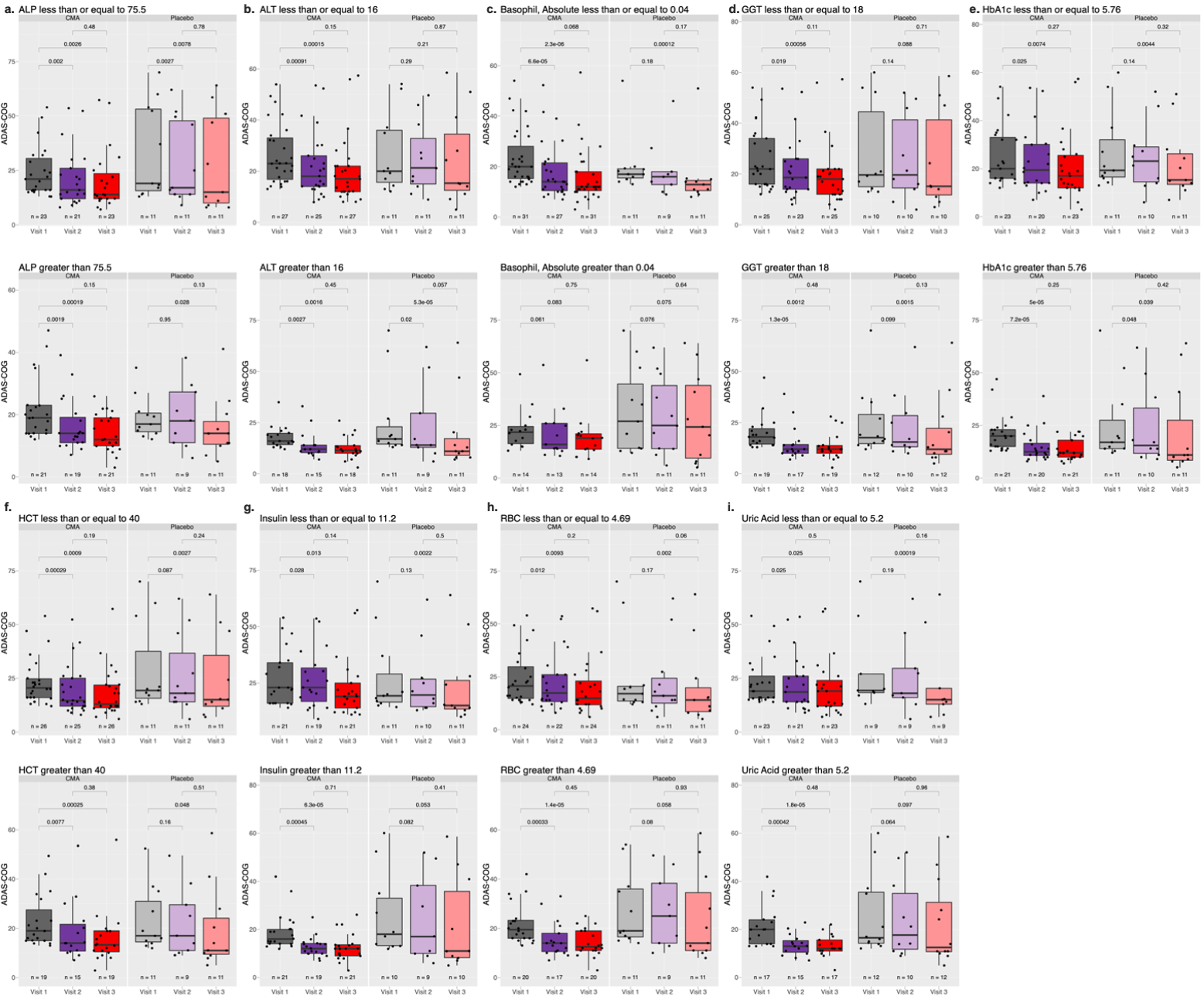

Supplement: Supplementary file 5 — Additional file 5: Fig. S2 Interindividual variability in clinical measures in responses to CMA administration. [file 40035_2023_336_MOESM5_ESM.tiff]
